# ‘Am I really the priority here?’: help-seeking experiences of university students who self-harmed

**DOI:** 10.1192/bjo.2023.652

**Published:** 2024-02-01

**Authors:** Alice Tickell, Peter Fonagy, Katalin Hajdú, Sandra Obradović, Stephen Pilling

**Affiliations:** Department of Clinical, Educational and Health Psychology, University College London, UK; School of Psychology and Counselling, Faculty of Arts and Social Sciences, The Open University, UK

**Keywords:** Self-harm, qualitative research, university students, help-seeking, service use

## Abstract

**Background:**

Self-harm is a significant problem in university students because of its association with emotional distress, physical harm, broader mental health issues and potential suicidality. Research suggests that fewer than half of students who have self-harmed seek professional help when at university.

**Aims:**

This study aimed to explore the help-seeking journeys of university students who had engaged in self-harm, to identify perceived facilitators and barriers to securing both formal and informal support.

**Method:**

Participants comprised 12 students who had self-harmed during their university tenure. Engaging in two semi-structured interviews over the academic year, they shared insights into their help-seeking behaviours and proposed enhancements to local services. Data underwent reflexive thematic analysis within a critical realist framework.

**Results:**

The analysis identified four themes: ‘The initial university phase poses the greatest challenge’, ‘Perceived criteria for “valid” mental health problems’, ‘Evading external judgements, concerns and consequences’ and ‘The pivotal role of treatment options and flexibility in recovery’.

**Conclusions:**

Students felt isolated and misunderstood, which amplified self-harming tendencies and diminished inclinations for help-seeking. A prevalent belief was that for self-harm to be deemed ‘valid’, it must manifest with a certain severity; however, concurrent fears existed around the ramifications of perceived excessive severity. Participants expressed a desire for streamlined pathways to mental health resources, encompassing both university and external mental health services. Insights from this study could guide future research and inform current service paradigms within academic and healthcare systems.

A growing body of evidence speaks to the problem of self-harm in university students.^1–3^ Estimates suggest that at least 7–23% of university students have self-harmed at least once, with 75% of these self-harming more than once.^1–3^ In this paper, self-harm is defined as any act of self-injury or self-poisoning, with suicidal, non-suicidal, mixed or unclear intent.^4^ People who have self-harmed often require mental health support to manage associated mental health problems and emotional distress, and to reduce the risk of suicide.^5^ However, fewer than half of university students who have self-harmed seek professional help.^6–9^ A recent study found prevalent negative views toward clinical services among those who self-harm, and noted minimal changes in the clinical management of self-harm over the past 16 years.^10^ Nevertheless, university students represent a distinct group with different social and professional connections and access to mental health services compared with the wider population. Therefore, it is crucial to understand the experiences of university students who have self-harmed, to understand if they report a similar pattern of dissatisfaction in mental health support.

## Summary of extant qualitative research

Current qualitative research indicates that university students see several advantages in seeking formal help for self-harm. These benefits include obtaining a diagnosis, referrals for therapy, learning to manage emotions and gaining a better understanding of themselves.^11^ Additionally, informal support from family and friends is valued for providing a supportive and understanding environment.^11^ However, students face both personal and social obstacles when seeking help. These include a wish to continue self-injuring, feelings of embarrassment and shame, fear of stigmatisation and concern about burdening others.^11,12^ Challenges specific to formal support are also reported, such as long waiting times, difficulties in accessing services, understaffed facilities, a lack of tailored and cooperative care and perceptions of self-harm not being serious enough.^11–14^

## Rationale for the current study

Existing qualitative studies have often adopted a more limited scope, focusing on specific aspects like students’ experiences of self-harm disclosure rather than proactive help-seeking for support, or their perspectives of clinical interventions for self-harm.^13,14^ There is a pressing need to dive deeper into the comprehensive help-seeking narratives of students who have engaged in self-harm within the university environment. This encompasses their interactions with professional services, peers and university staff. Addressing this gap, our study conducted interviews with students from a UK university who had self-harmed, aiming to understand their help-seeking journeys. In shedding light on this, we unveil a fresh perspective on the interconnected nature of support systems in the academic setting. This insight could pave the way for a cohesive, multifaceted approach to addressing students’ mental well-being. By shaping service delivery models rooted in students’ perspectives, we can enhance the identification, assessment and treatment of self-harm, ultimately promoting effective treatment engagement and recovery.

## Method

### Design

This study gathered data from students who had self-harmed when studying at a UK inner-city university. The design was shaped by a steering group of six students at the same university, with a history of mental health problems and self-harm, to maximise the relevance and acceptability of the project. They were consulted on the project aims and methods, including the content of questionnaires and interview schedules.

The researchers aimed to recruit 10–15 participants, seeking to generate comprehensive data to elucidate the multifaceted narratives surrounding students’ help-seeking experiences.^15^ Data collection consisted of an online questionnaire, followed by a semi-structured interview conducted via videocall (1.5 h), at two time points (3 h in total) taking place 3 months apart, in April (time point 1) and July (time point 2) 2021. The structure was designed taking into consideration the sensitive nature of the topic on self-harm. The questionnaires provided participants an avenue to communicate sensitive information in writing, paving the way for in-depth discussions during the interviews. Conducting interviews at two separate points ensured ample time for students to explore sensitive topics. This approach facilitated the collection of robust data on help-seeking from diverse sources, and captured the evolving experiences across the academic year, considering fluctuating university pressures and the availability of support.^16^ Each student was compensated for their participation.

### Participants

#### Recruitment

This study used a subset of data from the Journeys project, which gathered a large qualitative data-set on student self-harm at the university. The Journeys project recruited students from two mental health surveys conducted at the university in 2019–2020: the SENSE, and IMPACTS studies. In these surveys, those who had indicated recent self-harm when at university (‘Have you hurt yourself on purpose in any way in the past 12 months (e.g. by taking an overdose of pills or by cutting yourself?’)) and had agreed to be contacted for future research were emailed information about the project (*N* = 203). As the previous surveys were conducted in 2019–2020, some of these students may have already left the university. Of those who indicated interest (*n* = 39), the researchers checked the following eligibility criteria: 18 years old or over, current student at the university and residing in the UK. Twenty-five students were recruited on a first come, first served basis, by authors A.T. and K.H. This study analysed the subset of the data-set consisting of participants interviewed by author A.T. (*n* = 13), to retain researcher reflexivity throughout the analytic process. At time point 1, one student dropped out before interview as they said it would be difficult to discuss their mental health (*n* = 12). At time point 2, one student said they forgot to attend and then did not respond to subsequent contact (*n* = 11).

#### Characteristics

The 12 participants were mostly undergraduate UK fee-paying students. The majority were White cisgender women who identified as non-heterosexual. Almost all students reported that their mental health problems and self-harm began before starting at university. All participants had engaged in self-harm during their time at university, with half having self-harmed in the previous 3 months. Throughout the study, there were no reported suicide attempts, indicating that the self-harm during this period was non-suicidal. However, two students did disclose past suicide attempts. Although some students experienced suicidal thoughts during the study, they clarified that their self-harm was not motivated by suicidal intent. Across their lifetime, they had all received some type of psychological intervention, and almost all had taken psychiatric medication. Their full demographic characteristics, mental health history, self-harm behaviours and service use are summarised in Table 1.

**Table 1:** Participant characteristics

Characteristic	*n* (%)
Level of study	
Undergraduate	8 (67)
Graduate	4 (33)
Fee status	
UK fee-paying	11 (92)
Overseas	1 (8)
Ethnicity	
White British or European	7 (58)
Asian or Asian British	2 (17)
Mixed ethnicity	1 (8)
Black British	1 (8)
South American	1 (8)
Gender	
Cisgender woman	10 (83)
Transgender man	1 (8)
Non-binary person	1 (8)
Sexuality	
Bisexual	6 (50)
Heterosexual	4 (33)
Lesbian	1 (8)
Questioning	1 (8)
Onset of mental health problems	
Before university	11 (92)
During university	1 (8)
Self-reported mental health problems	
Anxiety or anxiety disorder	12 (100)
Depressive disorder	11 (92)
Eating disorder	5 (42)
Emotionally unstable personality disorder	1 (8)
Bipolar disorder	1 (8)
Recency of self-harm	
Past 3 months	6 (50)
Past year	2 (17)
Past 2 years	4 (33)
Onset of self-harm	
Before university	10 (83)
During university	2 (17)
Number of self-harm incidents since starting university	
1–5 times	3 (25)
5–10 times	4 (33)
10–20 times	1 (8)
≥20 times	2 (17)
Not sure or prefer not to say	2 (17)
Self-reported methods of self-harm	
Cut, scratched or stabbed self	10 (83)
Punched or hit self	3 (25)
Burnt self with fire or hot object	2 (17)
Choked self or tied ligature	2 (17)
Lifetime mental health service use	
Received counselling or therapy	12 (100)
Taken psychiatric medication	11 (92)
Experience of counselling/therapy	
Positive	7 (58)
Neither positive nor negative	3 (33)
Negative	1 (8)
Experience of taking medication	
Positive	3 (25)
Neither positive nor negative	3 (25)
Negative	5 (42)

### Ethics statement

The authors assert that all procedures contributing to this work complied with the ethical standards of the relevant national and institutional committees on human experimentation and with the Helsinki Declaration of 1975, as revised in 2008. All procedures involving human participants were approved by author A.T.'s university research ethics committee (reference number 16733/003). Informed consent was obtained electronically from all participants before data collection. The study employed a risk protocol to assist students at risk of harm (see Supplementary Appendix 1 available at https://doi.org/10.1192/bjo.2023.652). This protocol was activated once when a participant disclosed past suicidal thoughts and uncertainty about accessing support. The interviewer responded by directing them to suitable support services for potential future needs. All researchers with participant contact were trained in risk management and received regular supervision from a clinical psychologist.

### Materials

Study materials were based on existing questionnaires and interview protocols used in the IMPACTS study, adapted in consultation with the steering group to reflect their key concerns in relation to the topic of help-seeking for self-harm at university.

#### Online questionnaire

Demographic data were collected in the questionnaire administered at time point 1. The questionnaires administered at time points 1 and 2 also gathered clinical data, including information about current and historical mental health concerns, service use and frequency and method of self-harm (see Supplementary Appendix 2).

#### Semi-structured interview

Author A.T. conducted the interviews. She is a White cisgender woman, and her occupation was a trainee clinical psychologist, which meant that she was a doctoral student and an employee of the National Health Service (NHS). The interviews were structured with a topic guide, including questions on students’ self-reported facilitators and barriers to seeking support for their mental health and self-harm, and suggestions for improvements to mental health services (see Supplementary Appendix 3). Students were prompted to think broadly about help-seeking, including family, friends, academic staff, health professionals in the NHS, private healthcare and helplines. The interviewer received training in conducting interviews and pilot-tested the topic guide with members of the wider research team.

### Data analysis

The sample's demographics, mental health history, self-harm behaviours and service use were summarised. Interview data were audio-recorded, transcribed and anonymised. Transcription was jointly carried out by author A.T. and outsourced to members of the wider research team. Author A.T. analysed the interview data by using reflexive thematic analysis (RTA) to identify and report themes in the data within a critical realist framework.^17,18,19^ RTA was chosen as the analytic focus was on developing themes across the participants, rather than focusing on unique details of each case.^20^ The analysis was a broadly inductive and semantic process, meaning that coding was data-driven and not explicitly influenced by existing theories. Therefore, the analysis reflected students’ accounts of help-seeking as well as acknowledging the researcher's position (see Supplementary Appendix 4). Interviews conducted at time points 1 and 2 were analysed as one data-set, as the purpose of including two time points was to gather more in-depth data, and the research question did not seek to compare differences between time points 1 and 2.

Following the steps of RTA, author A.T. read the transcripts and took notes of initial observations of trends in the data. The data were uploaded to NVivo 12 for Windows (Lumivero, Denver, USA; see https://lumivero.com/products/nvivo/) to begin the preliminary coding process. The author A.T. worked systematically through the whole data-set, to code aspects of data items relevant to the research questions. Then, the third author coded a transcript from the data-set, and these codes were compared to encourage reflexivity. Author A.T. considered how the codes might combine to generate themes. These candidate themes were reviewed by the co-authors for internal consistency, ensuring there were clear and identifiable distinctions between themes. Based on this discussion, author A.T. wrote up an analysis for each theme, selecting illustrative quotations to represent each one. This was an iterative process, moving back and forth between the phases.^19^ Quotations were linked to randomly assigned identification numbers to protect participants’ anonymity.

## Results

The analysis of the interviews at time points 1 and 2 generated four themes (see Table 2) related to seeking help for mental health difficulties and self-harm when at university. The themes encompass self-reported facilitators and barriers to seeking support, and suggestions for improvements to mental health services.

**Table 2:** Summary and description of themes

Theme	Description
The initial university phase poses the greatest challenge	Starting university was often the hardest stage of help-seeking, as students had to establish new systems of informal and formal support alongside balancing the demands of student life and the transition to adulthood
Perceived criteria for ‘valid’ mental health problems	Some students felt their mental health problems were not valid enough to ask for help from overburdened mental health services, where the severest presentations were prioritised for support
Evading external judgements, concerns and consequences	Students shared information about their mental health and self-harm selectively to try and control the impression other people formed of them, to reduce the impact of stigma and to maintain autonomy
The pivotal role of treatment options and flexibility in recovery	Students wanted their needs and preferences to be at the heart of decisions around mental health treatment, and felt that this was instrumental in their recovery

### The initial university phase poses the greatest challenge

Most students started self-harming before university and had established support around this (including family, friends, school and health professionals). Beginning university often involved leaving home and relocating to another part of the country, living alone, often for the first time, and transitioning to adult mental health services. This made it difficult for students to seek help for self-harm, as they needed to establish new systems of informal and professional support alongside balancing the new demands of being at university. Those arriving from school perceived a sharp decline in support for their mental health:

‘I get that we're adults, so it's good to be less hand-holdy, but […] I don't think that means people should just be running loose and having no emotional support […] leaving home for the first time and then they're just like “OK, deal with it”’ [participant 2, woman, undergraduate].

At first, many of the students reported feeling lonely and socially isolated: ‘I very much felt like one in about a million, and no one knew who I was’ [participant 10, woman, postgraduate]. Some students felt that social isolation exacerbated their self-harm, as they could spend days in their room without anybody checking up on them, which meant that self-harm could go unnoticed. Students felt they needed more personal motivation to seek support, rather than having others notice a problem and proactively offering support (e.g. parents or teachers). However, they said their mental health problems drained their confidence, energy and motivation to seek support for themselves. They said their personal tutors were allocated by the university as a consistent point of contact, but many did not proactively ask them about their mental health:

‘Everything is very academic focused […] It's a question of […] Do you want to support students in their studies, or do you want to support students full stop?’ [participant 7, woman, undergraduate].

It was appreciated when staff asked directly about students’ well-being and created a sense of community through events and programmes: ‘I think that idea of like building a community is like very important beyond just like getting psychological help’ [participant 12, non-binary, undergraduate].

Students were not familiar with what mental health support was available and would ‘gossip’ and exchange negative stories about services (e.g. ‘I heard that you could only get an appointment if you were suicidal’ [participant 2, woman, undergraduate]), which discouraged help-seeking. Once students accessed support, it was common to experience a lack of integration between services. When students were signposted or referred elsewhere, they felt that their help-seeking journey ‘trailed off’ at this point, leading to deterioration:

‘Being shipped from person to person and referral to referral saying they don't know what's wrong with me […] All the referrals like that took another 6 months. And in between that time it was […] lots of self-harming. I had kind of an attempt and like lots of, massive mood swings, really low, low suicidal moods’ [participant 6, woman, undergraduate].

Students wanted information about mental health problems and services to be delivered during mandatory university contact. They called for more integration between the staff involved in their care (including general practitioners (GPs), university staff and mental health professionals) to streamline the process of help-seeking, to ‘put everyone in the same room with my questions and […] figure it out’ [participant 1, woman, undergraduate]. Students suggested having university staff with healthcare backgrounds as ‘bridges’ between the university and healthcare systems.

### Perceived criteria for ‘valid’ mental health problems

A pervasive barrier to help-seeking was students’ belief that their mental health problems were not valid enough to warrant support. They dismissed their difficulties as ‘normal student stress’ that others were dealing with too. Some students felt that university staff would only take them seriously if their mental health problems affected their academic performance. Students also reported similar concerns in relation to NHS providers, and believed they would only receive support if at risk of serious physical harm. This belief was influenced by perceptions of both university and NHS mental health services as underfunded. Students said they feared taking the place of someone who needed help more:

‘Things are bad, but […] I'm not actively standing on the edge of a bridge. […] When I'm seeking help from like the NHS […] am I really like, you know, the priority here?’ [participant 10, woman, postgraduate].

Students’ attitudes were sometimes formed in the context of dismissive responses from healthcare providers, leading them to believe that their problems were not serious enough:

‘The doctors said that I'm fine, then I'm not that sick […] I was hoping that by getting worse, I would finally get help ‘cause I felt unwell. Which is actually what happened […] About 8 months after, I had to go to the hospital because of a beginning of the beginning of organ failure […] that's when they reacted’ [participant 1, woman, undergraduate].

Some students felt they needed to escalate their self-harming behaviour to prove that their problems were valid, and to communicate that they needed support:

‘Having gone to A&E [accident and emergency], or had something, had a bit of a crisis, gave the GP the push that they needed. It's sad that it had to be that way, that me going and having a conversation with them, and asking for help wasn't enough’ [participant 6, woman, undergraduate].

When interacting with the NHS, some students obtained psychiatric diagnoses, which made it easier to access specialist services and meant they could register as having a disability with the university. Although some students found this validating and felt like their problems were finally considered legitimate enough to warrant support, others found it off-putting and pathologizing to receive a medical diagnosis to their problems. Therefore, students described a balancing act whereby they felt their condition needed to be severe to be taken seriously, but they worried about the consequences of being seen as too severe and therefore pathologized by their problems.

### Evading external judgements, concerns and consequences

Students shared information about self-harm with their social networks selectively, to reduce discrimination from others and maintain autonomy over their actions. They were more secretive about self-harm relative to other mental health problems, because people were less understanding about this, and often gave hostile, fearful or overprotective responses:

‘It's completely demonised. […] It's not something I talk about at all. […] I could talk about my anxiety or my grief […] but I would never be like “I'm struggling with self-harm”’ [participant 6, woman, undergraduate].

Mostly, students felt self-harm served an important function in helping them to cope with difficult emotions. From this perspective, they viewed self-harm as a ‘good thing for me […] if no one else had an opinion on it […] [But] it like it really upsets people’ [participant 3, woman, postgraduate]. Self-harm was conceptualised as a consequence of bigger problems, rather than the problem itself:

‘The anxiety is what causes it, and so the anxiety is the real problem, in a way. The self-harm is a symptom, not the cause’ [participant 8, man, undergraduate].

Students said their partners and families expressed high levels of distress regarding self-harm, which was experienced as pressure to stop self-harming. This made them feel guilty and more likely to conceal it. They said it was invalidating when people focused conversations on stopping self-harm, rather than acknowledging their underlying distress:

‘I just wanted someone to be like “I understand why you're doing what you're doing, why you feel like you haven't got any other ways to cope, it's not, it's not, it's not the best way, but I understand why you feel that this is your only option”’ [participant 6, woman, undergraduate].

Students found it helpful to talk to professionals with more understanding of self-harm, who could support them to develop alternative coping mechanisms. Some were afraid of talking to their GPs, in case they got admitted into hospital or it affected the cost of their health insurance, in which case anonymous helplines were viewed as helpful. They also found it helpful to speak to their peers with mental health problems, who they felt could relate to their problems. Organised opportunities to talk to peers (e.g. group therapy) were viewed as a helpful safeguard to avoid unhealthy dynamics arising, such as triggering others to self-harm.

Students feared declaring mental health problems to the university because of uncertainties about confidentiality and how this might affect their future employment opportunities (in relation to mental health stigma). Occasionally, students said that they received unhelpful responses from the university in response to disclosures, such as pressure to drop out of university or being invited to compulsory ‘check-ins’:

‘They almost always say “Have you thought about interrupting your studies?” And I always think, “Yes I have thought about it, but that's not really gonna help me deal with the problem.” […] I want more help for dealing with being a student with it, rather than I'm either a student, or I have a mental illness’ [participant 5, woman, undergraduate].

### The pivotal role of treatment options and flexibility in recovery

Students wanted their preferences to be at the heart of decisions around mental health treatment, and felt that this was instrumental in their recovery. Most said that medication was easy to access from GPs, although there were barriers to accessing more than 12 sessions of psychological support within the NHS. Many students expressed frustration if they were not given a choice regarding the therapeutic modality, and were typically offered cognitive–behavioural therapy. Some students reached the end of their help-seeking journey when they felt they had exhausted all of the publicly available treatment options:

‘Is this just how it is? Is this how I'm going to be for my whole life? It's not like I'm going to get a second life where I can do things properly, this is it. But then I have no way of changing […] Unless I pay from my savings for private therapy, which might not be effective’ [participant 4, woman, postgraduate].

Similarly, the university counselling service offered up to six sessions. Some students found this a helpful start to their help-seeking journey, but said it did not resolve their problems:

‘It was really great for […] like short term […] stress. But didn't get to the root of anything. Because there just wasn't enough time’ [participant 8, man, undergraduate].

They said that longer-term therapy would help them to ‘get to the root’ of problems once and for all. The university often signposted students to private practice psychotherapy to access longer-term support, rather than the NHS. This was only affordable if parents were able to pay for it, and students often felt guilty for asking their parents for financial support. One student reflected on how long they had struggled without access to long-term treatment from the NHS, before resigning themselves to paying for it privately with the support of family:

‘I think it is frustrating I just feel like you know, I've been dealing with these like issues for 7 years and I kind of feel like if I had just had access to long-term therapy like 7 years ago, I think things would be a lot different’ [participant 3, woman, postgraduate].

Students wanted flexibility on whether therapy was available online or face to face. For some, online therapy was a barrier to engagement if they did not have a private space at home, whereas for others, it was more accessible to fit in around a student lifestyle. However, others felt that online therapy was useful over the summer, when students may ‘never be in one place for long enough’ [participant 12, non-binary, undergraduate] to engage with face-to-face support from services.

## Discussion

### Summary of clinical, research and policy implications

#### Loneliness and constructing new social networks

Beginning university is often a difficult transition, as students must construct new social networks. Loneliness is a strong predictor of mental distress in students, and many find it challenging to form meaningful connections without pre-existing structures.^21,22^ This may be harder for students with prior mental health difficulties, as it can affect their motivation and confidence. Research is lacking in terms of what strategies are effective in addressing loneliness in students. Further studies are needed to understand this in different university contexts.^23^ According to student accounts, social isolation exacerbated self-harm, as they remained in their rooms without being noticed. To mitigate this, students felt that tutors should take a proactive role in inquiring about mental health, rather than solely focusing on academic matters. This reiterates a sector-wide uncertainty surrounding the responsibilities of academic staff regarding student mental health.^21^ Academic staff have reported not feeling equipped with the necessary training or time in their job plans to provide sufficient mental health support to students.^21^ Therefore, universities need to clarify what is expected of academic staff and allocate resources to support with any extra responsibilities, to ensure that students receive more consistent and equitable support.

#### Simplifying routes to professional support

Students experienced difficulties in forming networks of professional support because of the complexity of navigating different options across the university, NHS and private sector. This supports the advice of previous studies to simplify the route for accessing support at university.^24^ The heterogeneity of people who have self-harmed (ranging from those experiencing situational to enduring patterns of distress) necessitates careful assessment and matching of services to individual needs. This suggests the importance of clearer pre-university entry communication, to link students with prior mental health problems with appropriate support. Students deemed mental health promotion to be a crucial part of the university curriculum, including sessions on identifying mental health problems and seeking assistance. Weaknesses in signposting between services were highlighted as a vulnerable point in students’ help-seeking journey, and could be improved by establishing partnerships between university and local NHS providers (e.g. direct referrals between teams, sharing assessment protocols). Universities could additionally consider creating roles for staff with a healthcare background to provide advice for students struggling to access mental health services; this is in line with recent suggestions to hire university staff with mental health expertise, who are familiar with the context, language and systems of health systems.^21^

#### Balanced conversations about self-harm and safety

In line with a previous study, some students did not seek help for self-harm because they felt that it was helping them to cope with other issues such as depression, anxiety, interpersonal difficulties or stress.^14^ They believed that if they were not self-harming with suicidal intent, then self-harm was not serious. As a result, those who sought help presented at all types of services for other issues, without necessarily disclosing self-harm or seeking help to stop. They found it unhelpful when people excessively focused on risk, thereby pressuring them to stop self-harming, which caused them to be more secretive. Nevertheless, even self-harm that is not intended to be suicidal is associated with physical harm and an increased likelihood of future suicide attempts.^25^ Therefore, professionals need to take the students’ concerns seriously, but not focus excessively on risk assessment. Students said they wanted to receive non-judgemental support, with an emphasis on learning skills to manage difficult emotions. In this way, they felt that self-harm would decrease without needing direct intervention to stop self-harming. This aligns with current guidance on managing self-harm stating that healthcare providers should focus on safety planning instead of risk assessment.^26^

#### Holistic responses to university stress

Many students in this study dismissed their problems as ‘normal university stress’, which is a commonly reported obstacle to obtaining help from student populations, including those with suicidal thoughts.^27,28^ As half of students who self-harm do not receive any kind of professional help, it may be beneficial to provide entire student populations with interventions that teach stress management skills, with built-in systems to assist people in seeking treatment when needed.^7–10^ Universities could also take a holistic approach to stress, thinking about which aspects of university culture foster meaningful and challenging activities, and which aspects create unhelpful levels of stress that weaken students’ sense of confidence and competence.^21^

#### The importance of feeling understood

Students felt that their mental health experiences were not understood, which discouraged them from seeking help. Mirroring previous research, we found that students were selective about who they spoke to about self-harm, for fear of being misunderstood or judged.^11,13–15^ This applied to informal support, NHS and university staff, whose responses appeared to influence students’ negative perceptions of their own difficulties. To address this issue, some universities have implemented stigma interventions to educate the student body about mental health stigma.^29^ However, the results from this study suggest that to effectively reduce mental health stigma, a more bottom-up approach needs to be taken, which involves university and healthcare staff listening to the experiences of students. Historically, those receiving mental healthcare have often been denied agency in how they are supported, which has led to responses that were potentially harmful or ineffective.^21^ This study suggests that feeling heard and understood by academic staff and healthcare providers could facilitate help-seeking behaviour. This provides a rationale for involving students in shaping mental health strategies, with varying levels of involvement from consultation to co-production.^30^

Students noticed that the mental health services were overburdened, with long waiting lists, limited number of sessions offered and a lack of treatment choices. This led them to feel like they were not a priority, and that there were others who need the support more. This led students to disengage from help-seeking, or to have a ‘crisis’ to prove their need for help. This highlights the need to better resource existing services to provide person-centred support to more people who have self-harmed, even if there is no perceived risk to their immediate safety.

### Limitations and future directions

The study's recruitment process, which targeted students for their perspectives on seeking help for self-harm, attracted only a small fraction of the people invited to participate. This may reflect the inherent challenges in discussing self-harm, as evidenced by one student withdrawing before the study because of the anticipated difficulty in discussing their mental health. Consequently, the study sample may disproportionately represent students who are particularly motivated to enhance mental healthcare or more comfortable discussing their mental health issues. In the current sample, all students had sought professional help for their mental health when at university; therefore, help-seeking was more prevalent in this sample than in the larger population of students who have self-harmed.^7–10^ Students who do not seek help may experience more pronounced barriers to their help-seeking, coupled with fewer facilitators, or may face additional barriers not identified by this study. Future research could benefit from purposively sampling those who have not sought help for their difficulties.

The sample was predominantly women (83%) and identified as non-heterosexual (67%). This could reflect the fact that women and sexual minorities are at increased risk of self-harm.^31,32^ Evidence suggests that LGBTQIA+ students are less likely to access traditional services, so it is useful to have captured their voices in this study.^29^ In addition, most of the sample were White (58%) and cisgender (83%). Other similar qualitative studies on self-harm also reflect this demographic skew in their samples toward White cisgender women, so this a broader limitation in the field of self-harm research.^13–15^ Future studies could purposively sample student groups who are relatively underrepresented in the current sample.

This study explored the reasons for low levels of help-seeking in students who had self-harmed when at university.^7–10^ This could generate new means of service delivery, which may be more accessible than services designed solely by professionals.^21,24^ Students called for more scaffolding, particularly at the start of university, to establish social and professional networks of support, including holistic mental health education integrated into the university curriculum. They called for more proactive interest and understanding from university staff regarding their mental well-being. They wanted their mental health problems to be taken seriously, regardless of risk level, to catch problems early and prevent them from escalating. Students felt that it was crucial for professionals to treat them as the experts in their own problems, and listen to them regarding their treatment choices to support their recovery. Future research should focus on empirically testing the recommendations from this study within university settings and the NHS framework. Additionally, it will be vital to include the perspectives of individuals who care for students who self-harm. This approach will ensure a comprehensive understanding, and enable the development of more effectively tailored support networks by incorporating insights from all relevant stakeholders.

## Supporting information

Tickell et al. supplementary material Tickell et al. supplementary material

## Data Availability

The data are not publicly available due to their containing information that could compromise the privacy of research participants.
