# Supplementary material for: ‘Am I really the priority here?’: help-seeking experiences of university students who self-harmed
Source: BJPsych Open. 2024 Feb 1;10(2):e40. doi: 10.1192/bjo.2023.652 (PMC10897682; doi:10.1192/bjo.2023.652)
Supplement: Tickell et al. supplementary material [file S205647242300652Xsup001.docx]

**Supplementary Materials**

**Supplementary Materials 1: Risk Protocol and Mitigation**

Risk issues that might arise from the study and how they will be addressed:

1. Participant distress and/or disclosure of serious harm or risk during interviews/free-text online survey responses

Due to the context of the interviews and online surveys, some content will cover questions on students’ presenting mental health problem and their reasons for seeking help.

**Mitigation**

While there is a chance that some participants may find these questions distressing, during this process participants will be reminded that they can stop the interview/ online survey, leave out any questions they do not wish to answer, or withdraw from the study at any time. Participants will have a point of contact in the team (a “participant case manager”) who will conduct their interviews and send requests for online data in-between interviews. Participant case managers will be in clinical training (being Trainee Clinical Psychologists), will be trained in risk management, and will have weekly supervision, during which any issues will be discussed. Trainee Clinical Psychologists are NHS employees and professional doctoral students, who have extensive training in working clinically with mental health problems and with risk management. In addition, the interviews and online surveys will be developed in collaboration with experts-by-experience, who will be asked whether they think the questions are appropriate or whether they may be upsetting for some students. If a participant indicates that they are in risk of harm to themselves, appropriate action will be taken in line with risk protocols. This specifies that if a participant indicates they are at serious risk (e.g., suicidal thoughts or plans that are not deemed immediate by the case manager), then the case manager will discuss this further with the participant, offer signposting including crisis support available, document the discussion, and monitor progress closely. If a participant is deemed to be in immediate serious risk (e.g., suicidal thoughts and plans that are deemed to be immediate by the case manager), additional action will be taken which may include contacting the participant’s GP, Crisis Resolution and Home Treatment Team, or completing a Student of Concern form. In any instance where there is deemed to be any risk to the participant (minor or serious), this will be discussed during weekly supervision meetings with a qualified clinical psychologist. Participant case managers will be responsible for being responsive to potential causes of concern, and any decisions about actions will be taken with their supervisor/ the study PI. It will be made clear to participants that the case managers will not be able to provide direct clinical support and that they are not clinical advisors. Participant case managers may signpost to services that reflect the needs of the participant, such as university mental health coordinators within the Student Support and Wellbeing Service (this is the most likely service which would be relevant for students who are struggling to navigate the system).

1. The possibility of participant distress and/or disclosure of harm during standardised questionnaires

The questionnaires that will be asked will cover student mental health, self-harm, online activities, and risk factors for care barriers. There could be a possibility that participants become distressed when answering questions about their mental health.

**Mitigation:**

Only standardised mental health symptom questionnaires, which have been validated will be used. Participants will be reminded that they can leave out any questions they do not wish to answer and are free to withdraw at any time. If a participant indicates they are in serious risk of harm to themselves, they will be invited for a brief phone call with their participant case manager, to assess their risk and discuss resources available.

1. Disclosure of problems with current treatment or care

As the study will be recruiting students who are currently seeking or receiving care from a service, there is a possibility they will be experiencing problems with their treatment or care at a time of data collection. This incorporates a range of situations, from malpractice on the part of a service leading to serious risk, to situations which carry less immediate risk (e.g., administrative difficulties experienced by international students).

**Mitigation:**

If a student discloses in their interview or online survey that they are experiencing problems with their current care that would lead to serious risk or harm, the participant case manager will first discuss this further to try and understand more about the issue, before reporting it to the PI, and documenting the discussion. Participants will be signposted to relevant advice services (i.e., drop-in session with university mental health coordinators) to support them in exploring their options moving forward.

1. Disclosure of confidential information

Participant case managers will discuss any concerns they have about the safety of participants, or other people, with the Principal Researcher during their weekly supervision sessions. The limits to confidentiality are if the participant case manager and Principal Researcher believe there to be significant risk to the safety of the participant (e.g., repeated self-harm which is not already being managed by their GP; or suicidal plans) or to anyone else. In this case, a decision will be made about whether disclosure of information to the participant’s GP, another relevant NHS service (e.g., Crisis Resolution and Home Treatment Team if there is urgent risk), University Student Support and Wellbeing (via a Student of concern form) or, in rare situations to other external agencies (e.g., the police) is appropriate.

**Mitigation:**

Information will only be shared with participants’ GPs if they have been assessed by the Principal Researcher and participant case manager to be at risk of harm to themselves or others. Only information which is immediately relevant to ensuring the safety of the participant/ any others will be shared. In the majority of cases, it is expected that the participants’ GP will be the relevant person to speak to. In some cases, it may be necessary to speak to Student Support and Wellbeing, using a Student of Concern form. Where this is the case, particular care will be taken to ensure that only information relevant to the participant’s safety is shared. The participant case manager will discuss the confidentiality policy with each participant in their initial interview, to ensure they have a good understanding of the circumstances under which a disclosure might be necessary, and they will make it clear that participants can contact them at any time if they wish to discuss this further. The participant case manager will always seek to discuss any disclosure with participants before doing so.

1. Potential intervention to support students to access care

An important factor in students’ accessing appropriate care is their knowledge about available services. However, there is a potential conflict between the ethical duty to help support student participants through the provision of information and the objective of the research study of observing ‘natural’ behaviour and situations. In some cases, participant case managers may become aware of a problem with the mental health care being received by a student (e.g., it becomes apparent that a students’ referral has been lost by a service).

**Mitigation:**

In designing this study, considerable care has been taken to balance these two potentially conflicting objectives. It has been decided that it will not be appropriate to observe students’ behaviour and situations without intervening with the provision of information, where appropriate. This has been accepted by the research team as a limitation to the study. However, the participant case managers will take care to explain to participants that they are not clinical advisors, and in most cases, they will signpost the participants to a more appropriate person to advise (e.g., a University Mental Health Coordinator or their GP). Where participant case managers become aware of problems with a student’s care, they will discuss this in supervision with the Principal Researcher. The Principal Researcher (a Clinical Psychologist) will either be able to advise the participant about an appropriate course of action or intervene where appropriate, with their consent.

**Supplementary Material 2: Survey questions on mental health, service use, and self-harm**

The following questions ask about your mental health.

Have you ever had, or suspected that you had a mental health problem?

- Yes
- No
- I prefer not to say

Please provide more details about the mental health problems you have experienced:

[Free text response]

How old were you when you first experienced any mental health problem?

[Free text response]

Have you had any mental health problems while at university?

- Yes
- No
- I prefer not to say

Has a professional ever diagnosed you with a mental health problem?

- Yes
- No
- Not sure
- I prefer not to say

What was the diagnosis you received? If not sure, please also provide further details:

[Free text response]

The following questions about your use of services.

Have you ever had counselling or therapy for a mental, nervous, or emotional problem?

- Yes
- No
- Not sure
- I prefer not to say

Which type(s) of counselling or therapy did you have?

Select all that apply

▢ Psychotherapy or psychoanalysis

▢ Cognitive behavioural therapy

▢ Art, music, or drama therapy

▢ Social skills training

▢ Couple or family therapy

▢ Sex therapy

▢ Mindfulness therapy

▢ Alcohol or drug counselling

▢ Counselling (including bereavement)

▢ Drop-in support service

▢ One-off wellbeing workshop

▢ Not sure

▢ Another type of therapy (please describe): ______________________________

▢ I prefer not to say

What type of service did you receive this from?

Select all that apply

▢ University support service

▢ NHS service (or equivalent in home country)

▢ Private service

▢ Secondary school support service

▢ Charity

▢ Other (please describe): ________________________________________________

▢ I prefer not to say

How old were you when you received these services?

Please list if you have accessed multiple services

[Free text response]

How was this experience?

- Very positive
- Quite positive
- Neither positive or negative
- Quite negative
- Very negative

Please explain the reason to why your experience was positive/negative here:

[Free text response]

Have you ever taken any medication for a mental, nervous, or emotional problem?

- Yes
- No
- Not sure
- I prefer not to say

What was the type of medication that you had?

- Antidepressant
- Anti-anxiety
- Antipsychotic
- Other (please describe): _____________________________________________
- Not sure
- I prefer not to say

How old were you when you received this?

Please list if you have taken multiple medications.

[Free text response]

How was this experience?

- Very positive
- Quite positive
- Neither positive or negative
- Quite negative
- Very negative

Please explain the reason to why your experience was positive/negative here:

[Free text response]

The following questions ask about times you may have intentionally hurt yourself.

Have you hurt yourself on purpose in any way while at university?

- Yes
- No
- Not sure
- I prefer not to say

What did you do to hurt yourself?

Leave blank if you prefer not to say

[Free text response]

How old were you when you first remember doing this?

Leave blank if you prefer not to say

[Free text response]

How many times have you done this while at university?

If you are not sure, it is okay to make a rough estimate

- 0-5
- 5-10
- 10-20
- 20-50
- 50+
- Really not sure
- I prefer not to say

When was the last time you did this?

Leave blank if you prefer not to say

[Free text response]

**Supplementary Material 3: Interview Schedules**

**T1 Interview Schedule**

| **Question** | **Prompt** | |
| --- | --- | --- |
| 1 | | Firstly, I would like to start by talking about your experiences of mental health while at university.  Can you tell me about the times you have struggled with your mental health since starting at university? We could think of it a bit like a timeline. Can you give examples of specific times that you found particularly difficult? |
| Follow up/prompts | | - In your questionnaire, you said that you have experienced <anxiety/depression/eating disorder, etc.> while at university. Can you tell me about how that impacted you? |
| 2 | | Who knows about these difficulties? Who have you spoken to about it? |
| Follow up/prompts | | Encourage them to list everyone they have spoken to… Prompt:   - This could be a person from your family, a friend, academic staff, a therapist/counsellor, doctor, people online… - If they don’t mention it: You also mentioned in your questionnaires that you have received counselling/therapy/medication while at university.   Then explore each in more depth, in chronological order if possible   - Starting with <person>, can you tell me about your experience of talking to them?   Additional prompts:   - When did you talk to them? - Why then? How did the conversation come about? What prompted the conversation? - Did you approach them, or did they approach you? - What made you want to talk to them? What were you expecting? - What happened? Did it match your expectations? What was good/bad about it? - How did that make you feel? (About yourself/your problems?) - How did this affect you/ change things for you?   Who did you speak to next?   - Same as above   (If relevant) COVID-19 impact:   - How did the COVID-19 pandemic affect your experience of talking to them?   Prompt anyone else not mentioned:   - Is there anyone else who you have spoken to or tried to speak to about your difficulties?   If they have not spoken to anybody:   - What influenced your decision not to talk to anybody? |
| 3 | | So far, we have covered who you have spoken to about your difficulties while at university, and we will certainly discuss that in more depth as we go along.  Next, I would like to ask some questions about experiences of self-harm. Remember, you do not have to answer any of my questions, and you can tell me if you do not feel like answering any of them.  In your questionnaires you indicated that you have [self-harm method(s)] while at university, X times.  Can you tell me about the situations that led you to [self-ham method(s)] while at university? |
| Follow up/prompts | | Prompts:   - What would you say are the main triggers (for self-harm)? - Can you tell me about the times or situations where you were doing that more? - What about the times or situations where you were doing that less or not at all?   Prompt intention:   - When you do that, what is your intention? - [Has your intention ever been to end your life?] - How do you feel about the term ‘self-harm’? Do you feel like those words reflect your experience?   Prompt for their views:   - What effect does this have on you? - Is this a good thing or a bad thing? - Has it always been like that? Have you ever felt differently about it?   If they have mentioned more than one self-harm method:   - Do you do one method more than the other? - In what ways do the [self-harm methods] differ for you?   Expand on self-harm:   - Thinking about self-harm more broadly, is there anything else which you did not mention in your questionnaire that you have done even though you knew it would hurt you, or with the intention to hurt yourself? |
| 4 | | Before, we talked about who you have spoken to generally about your main difficulties, but I am interested to hear if there is anybody who knows about [self-harm]? (This could be a person from your family, a friend, academic staff, a medical professional in the NHS or a private service, a therapist or counsellor, somebody on a helpline) |
| Follow up/prompts | | Get them to list everyone they have spoken to… Then explore each in more depth, in chronological order if possible   - Starting with <person>, can you tell me about your experience of talking to them?   Intention:   - When did you talk to them? - Why then? How did the conversation come about? What prompted the conversation? - Did you approach them, or did they approach you? - What made you want to talk to them? What were you expecting?   Their intention/the impact:   - What happened? Did it match your expectations? - How did that make you feel? (About yourself/your problems?) - How did this affect you? Did that change things for you? How?   Who did you speak to next?   - Ask the same questions as above   (If relevant) COVID-19 impact:   - How did the COVID-19 pandemic affect your experience of talking to them?   Prompt anyone else not mentioned:   - Is there anyone else who you have spoken to or tried to speak to about the self-harm?   If not mentioned, prompt medical attention:   - Have you ever received medical attention for your [self-harm]? - How many times has that happened? - Ask questions above   If they have not spoken to anybody:   - What influenced your decision not to talk to anybody? - Have you thought about approaching anybody? |
| 5 | | Are there any other sources of support you know about that you think could be helpful for your difficulties? This could be anything we have not talked about yet – such as a person, helpline, online information, anything at all that you think might help you. |
| Follow up/prompts | | - What do you know about them? How did you find out about them? - Have you tried to get support there? Are you planning to get support from them? What has influenced that decision? - What would need to change for you to reach out? |
| 6 | | Thinking forward, what is coming up for you at university? When I next talk to you in 3 months or so, how do you think things will be for you? Do you anticipate any particular challenges? How do you plan to handle things going forward? |
| 7 | | Is there anything else you would like to say about your experience of seeking help for your mental health while at university?  How have you found it answering those questions? |

**T2 Interview Schedule**

| **Question** | **Prompts** |
| --- | --- |
| 1 | How has your mental health been since we last spoke to each other? |
| Follow up/prompts | Prompts:   - On your survey you mentioned that your mental health has been better/stayed about the same/gotten worse. Can you say more about that? - What contributed to your mental health being better/the same/worse? - How has the ongoing COVID-19 pandemic affected your mental health?   Self-harm:   - On your survey, you mentioned that you [self-harm method] X times. What do you think triggered that? What was the sequence of events? - What did you think after? How did you feel after? - Has anything changed about how you self-harm?   Or if they have not self-harmed:   - On your survey, you mentioned that you did not self-harm since we last spoke. Are you aware of anything that might explain this? Anything that happened or any changes for you? |
| 2 | Who have you been speaking to about your mental health since we last spoke to each other? |
| Follow up/prompts | Prompts:   - If they don’t mention it: You also mentioned in your questionnaires that you talked to [your GP/a therapist] - When? Why then? What prompted the conversation? - What made you want to talk to them? - What happened? What was good/bad about it? - How did that make you feel? - How did this affect you/ change things for you? - How did the COVID-19 pandemic affect your experience of talking to them?   Self-harm   - We have talked about who you have spoken to generally about your main difficulties, but I am interested to hear if there is anybody who you spoke to about your [self-harm] or [self-harm] in general? - If yes: use some of the prompts above - If no: see below   If they have not spoken to anybody:   - What influenced your decision not to talk to anybody? - How did the COVID-19 pandemic affect your experience? |
| 3 | So far, I have asked you about your recent experiences of your mental health and who you have spoken to about that.  Now I would like to ask you to reflect on your mental health journey as a whole since starting university.  Up until now, how much do you feel that you got the support you needed for your mental health? |
| Follow up/prompts | Prompts:   - What was the most helpful type of support you received? What was less helpful? - What got in the way of you getting the support you needed? What helped you to get the support you needed? |
| 4 | If you could take a blank sheet of paper, and design the ideal type of support you would have wanted for your mental health, what would it look like? |
|  | Prompts:   - Where and how would it be offered? How would you access it? - When would it be offered?   Self-harm:   - During your time at university, would you have ever wanted additional support to manage [self-harm/crisis]?   - What would that look like?   - Where/how would it be offered? How would you access it?   - When would it be offered? |
| 5 | Is there anything else you would like to say about your experience of seeking help for your mental health while at university? |

**Supplementary Material 4: Account of reflexive practice**

Throughout the research process, I engaged in the self-reflexive process of bracketing, where I brought awareness to my own perspective, pre-existing thoughts and beliefs, and developing hypotheses.^1^ The purpose of this was to critically reflect on the ways that I as the researcher may have affected the process.^2^ This included a bracketing interview, a reflexive diary, and reflective discussions with my supervisor, co-researcher, and the steering group.

At the time of conducting the study, I was a doctoral student at the same university as my participants. Therefore, I came to the study with my own experiences of being a student and engaging with university systems and support services. When I was an undergraduate student at a different university, I recalled experiences of friends struggling to access support for self-harm. This influenced my motivation to conduct the research, to give voice to students and influence systems to become more accessible. I expect that having a shared identity with my participants as a ‘student’ helped to foster an alliance between us, which enabled more open sharing of their experiences. However, I noticed my expectation that students would have had similar experiences to those that I had previously heard about at another institution. I reviewed my interview schedule to ensure questions were open, to capture all types of experiences. As well as being a student, I was a trainee clinical psychologist, which means that I believe that psychological approaches to supporting people are often effective and helpful. However, in my interviews I wanted to interrogate all aspects of help-seeking, not just psychological therapy. I discussed this with the steering group in advance, to gain a more balanced view of all the potential source of support that students may be interested in. During interviews and analysis, I brought curiosity to examples of successful help-seeking, alongside challenges.

**Supplementary Materials References**

1. Starks H, Trinidad, SB. Choose your method: A comparison of phenomenology, discourse analysis, and grounded theory. Qualitative Health Research. 2007;17(10):1372–80. <https://doi.org/10.1177/1049732307307031>
2. Braun, V. & Clarke, V. Successful qualitative research: A practical guide for beginners. United Kingdom: Sage; 2013.
